# Ventilation, oxidative stress and risk of brain injury in preterm newborn

**DOI:** 10.1186/s13052-020-00852-1

**Published:** 2020-07-23

**Authors:** Laura Cannavò, Immacolata Rulli, Raffaele Falsaperla, Giovanni Corsello, Eloisa Gitto

**Affiliations:** 1 Department of Human Pathology of Adulthood and Childhood, University of Messina, UOC di Pediatria, Pad. NI, 3° piano, AOU Policlinico Gaetano Martino, Via Consolare Valeria, 1, 98125 Messina, Italy; 2 Department of Human Pathology of Adulthood and Childhood, University of Messina, Messina, Italy; 3 Department of Paediatrics, Policlinico-Vittorio Emanuele University Hospital, Catania, Italy; 4 Department of Maternal and Child Health, University of Palermo, Palermo, Italy; 5 Department of Human Pathology of Adulthood and Childhood, University of Messina, Messina, Italy

**Keywords:** Oxidative stress, Ventilation, Brain injury, Preterm

## Abstract

Preterm infants have an increased risk of cognitive and behavioral deficits and cerebral palsy compared to term born babies. Especially before 32 weeks of gestation, infants may require respiratory support, but at the same time, ventilation is known to induce oxidative stress, increasing the risk of brain injury. Ventilation may cause brain damage through two pathways: localized cerebral inflammatory response and hemodynamic instability. During ventilation, the most important causes of pro-inflammatory cytokine release are oxygen toxicity, barotrauma and volutrauma. The purpose of this review was to analyze the mechanism of ventilation-induced lung injury (VILI) and the relationship between brain injury and VILI in order to provide the safest possible respiratory support to a premature baby. As gentle ventilation from the delivery room is needed to reduce VILI, it is recommended to start ventilation with 21–30% oxygen, prefer a non-invasive respiratory approach and, if mechanical ventilation is required, prefer low Positive End-Expiratory Pressure and tidal volume.

## Introduction

Birth before 37 weeks of gestational age (GA), defined as preterm, has an incidence of 7–12% worldwide [1]. Compared to infants born at term, preterm babies have an increased risk of acute and chronic brain injuries such as cognitive deficits and behavioral (40–50%) and cerebral palsy (5–10%) [2, 3]. The period of highest risk of brain injury is 23–32 weeks of GA.

Since 1999, inflammation, oxidative stress (OS) and hemodynamic pathways were known to play a critical role in the pathogenesis of brain injury in preterm infants [4, 5]. The high sensitivity of premature babies to oxidative damage is due to the imbalance deriving from the overproduction of free radicals and the insufficient levels of antioxidant enzymes, such as superoxide dismutase, catalase, glutathione peroxidase and glutathione reductase, especially before 32 weeks of gestation [6–8]. Several pharmacological treatments to mitigate the production of free radicals have been studied (i.e. therapy with maternal glucocortico-steroids and allopurinol, erythropoietin and melatonin, but an ideal therapeutic strategy has not yet been found [9–16].

Moreover, in infants before 32 weeks of gestation, lung disease may require non-invasive respiratory support (NIV) or mechanical ventilation, but on the other hand, oxygen administration since the delivery room is the main intervention that can cause OS. Consequently, any intervention applied perinatally, including respiratory assistance, must be optimized because it can increase the incidence of brain injury [17]. Nevertheless, almost 90% of premature babies who need assisted ventilation receive dangerously high volumes of air (VT), with potentially harmful effects on the lungs and the brain [18–20].

## Ventilation-induced brain injury in preterm neonates

To date, it is known that inflammation and systemic and brain injuries are often triggered by ventilation-induced lung injury (VILI) [4, 21]. Several studies demonstrated that ventilation may cause brain damage through two pathways: localized cerebral inflammatory response and hemodynamic instability. The first is due to the inflammatory pulmonary cascade that crosses the blood-brain barrier, causing pro-inflammatory cytokines overproduction and brain white matter injury [22]. Already after 2 h of ventilation, an increase in the pro-inflammatory plasma cytokines of interleukin (IL) -8, IL-1β and TNF- α and a decrease IL-10 anti-inflammatory cytokines have been demonstrated [7, 23]. An increase in pro-inflammatory cytokines can also compromise cerebral vascularization and reduce the integrity of the blood-brain barrier. The main reason of free radical overproduction is the use of pure oxygen during resuscitation in the delivery room. However, although there is evidence of an increase in OS when starting preterm infants in 100% oxygen, it is known that both hyperoxemia and hypoxemia should be avoided [24]. Indeed, the combination of heart rate < 100 / min and SpO2 < 80% in the first 5 min is associated with death or intracranial hemorrhage [25]. The second pathway is connected to the over-distension of the preterm alveoli, to the compression of the pulmonary capillaries and to the pulmonary hemodynamic instability which leads to an alteration of the pulmonary venous return and of the cardiac output and wide oscillations of the cerebral blood flow (CBF). Indeed, barotrauma caused by the application of intermittent positive pressure ventilation (IPPV) and Positive End-Expiratory Pressure (PEEP), induce the variability of intrathoracic pressure, which can affect preload, post-load, heart rate and myocardial contractility, altering the hemodynamic function of heart and brain. Moreover, some authors demonstrated that over-distention of the alveoli amplifies the pulmonary inflammatory response, which increase in the gene expression of pro-inflammatory cytokines in the brain of ventilated preterm lambs [26]. These paths are amplified by duration of ventilation and high VT [22, 27–29]. Indeed, concerning the volutrauma, some authors compared two groups of ventilated preterm infants with a VT < 5.8 ml/kg and > 5.8 ml/kg and described a higher incidence of intraventricular hemorrhage (IVH) in premature babies who received high VT compared to children who received lower VT in the delivery room (51% vs 13%) [20, 30]. Three large VT breaths are sufficient to initiate an inflammatory response, while lower VT improves cerebral hemodynamic stability and reduces the inflammatory response [28]. Finally, the increase in pro-inflammatory cytokines cause the widespread activation of microglia within the immature white matter, decrease the ability of cerebral vascularization to protect against abnormal CBF, as well as reduce the integrity of the blood-brain barrier, making it more prone to bleeding [23, 28]. Histologically, the damage of the cerebral parenchyma is evident with an increase in the size and density of the microglial aggregations [28].

Therefore, given the underlying risk of brain injury in premature babies, the need for ventilation further aggravates the likelihood of acute injury and chronic disability by suggesting the imperative that a premature baby receive the safest respiratory support possible from the delivery room.

## Ventilation protective strategies in the delivery room

Neonatologist are familiar with the concept that prolonged mechanical ventilation can be injurious to the preterm lungs and brain, and that a gentle ventilation is required to reduce VILI from the delivery room. It is also known that endotracheal intubation can damage premature lungs by triggering inflammation pathways, so NIV should be preferred and multiple ventilation strategies should be attempted to reduce injury and improve results.

An investigation comparing initial FiO2 of 0.30 vs 0.65 for preterm infants of about 1 kg, reported that clinical outcomes were equivalent [31]. However, other authors reported that an initial FiO2 of 0.30 during resuscitation of infants of GA < 28 weeks resulted in decreased oxidative stress markers [32]. Based on these data, the latest guidelines suggest starting premature infants < 28 weeks with approximately 30% oxygen, while infants between 28 and 31 weeks of gestation with 21–30% oxygen, until further evidence is available [24].

Peak inspiratory pressure (PIP) and VT are highly dependent on the respiratory device. According to recent surveys, the devices commonly used in the delivery room are self-inflating bags, inflatable (anesthetic) bags and T-resuscitators [33]. The main difference between T-piece resuscitators and self-inflating bags is that the former has pressure relief valves for PIP and PEEP, while self-inflating bags are often used without pressure gauges. Consequently, T-piece resuscitators provide accurate, reliable and well-controlled PIP and PEEP compared to inflatable bags [34, 35].

Due to the lack of adequate device feedback, resuscitators in the delivery room often cannot estimate VT and preterm infants risk receiving high VT [36].

## Ventilation protective strategies in the NICU

When infants only require a non-invasive respiratory approach, the two most common forms of NIV in neonatal intensive care units (NICU) are nasal Continuous Positive Airway Pressure (nCPAP) and High-Flow Nasal Cannula (HFNC). The nCPAP may be use to facilitate the initiation of spontaneous breathing and to reduce mechanical ventilation, preventing the adverse effects that can result from intubation [37]. Several studies have found that nCPAP reduces the need for supplemental oxygen, intubation, ventilator support median days, and postnatal corticosteroids, maintaining long-term respiratory benefits [28]. Today most tertiary units use nCPAP for the stabilization from the delivery room, but the optimal timing of CPAP remains receding [38, 39]. A popular variant of CPAP is Bi-level CPAP or BIPAP that uses small pressure differences between the inspiratory and expiratory phases. However, there is no evidence that BIPAP confers any advantage over CPAP. Another ventilation mode with nasal interfaces is high nasal frequency oscillatory ventilation (NHFOV), but the results have been inconclusive [25].

The administration of heated, humidified oxygen by HFNC is an alternative to nCPAP used between 55 and 80% worldwide [40–42]. However, its use as a primary respiratory support modality was inferior than CPAP in terms of failure, because infants randomized to HFNC often needed CPAP rescue to prevent intubation [43]. Clinical trials have shown that HFNC is substantially equivalent to nCPAP for infants > 28 weeks GA, but nCPAP remains the preferred initial method of non-invasive support, particularly for younger children [25].

Despite an initial optimal management with NIV, almost half of infants < 28 GA require mechanical ventilation and are therefore at additional risk for VILI and OS [44]. The purpose of mechanical ventilation is to inflate the atelectasis lung, optimizing the lung volume for a uniform distribution of the tidal volumes at set pressures to prevent atelectasis and over-distension. A ventilation strategy to reduce inflammatory lung injury is to obtain a recruited or “open” lung with an adequate VT by adjusting the optimal PEEP level for each patient.

Traditionally, infants’ ventilators were used in a limited pressure ventilation (PLV) mode, but new volume targeted ventilation (VLV) modes have been developed to reduce VILI by setting the amount of air entering the lungs with each inflation. Recently, a Cochrane comparing VLV versus PLV in infants showed that the use of VTV reduced the rates of serious brain disease, i.e. IVH grade 3 or 4, or periventricular leukomalacia (PVL), or both. Probably, it is due to the fact that the VTV modes, by controlling the VT, avoid volutrauma, improve the stability of the parameters of the gases in the blood and reduce hypocarbia, stabilizing cerebral perfusion and reducing neonatal brain lesions [45].

Another ventilation strategy is guaranteed volume (GV) mode which corrects inspiratory pressure giving a fixed current volume based on changes in compliance, resistance and spontaneous activity to limit lung volutrauma [46]. Some data suggest that this strategy may reduce the acute inflammatory response, and thereby also limit oxidative stress in preterm infants [47].

Finally, in the last few decades, newer microprocessor controlled ventilators with ever improving capabilities for synchronization of breaths, adjustment for gas leaks, accurate measurement of small tidal volumes, and automation of adjustments of peak inspiratory pressure, rate, and inspired oxygen were introduced. As the use of newer technologies, such as artificial intelligence and machine learning, are gradually incorporated, it is possible that they will help improve clinical decision making in order to reduce damage of ventilation related oxidative stress, including the brain injury.

## Conclusions

Inflammatory pathways are known to play a key role in the pathogenesis of brain injury through direct toxicity induced by gliosis or through greater permeability of the blood-brain barrier, as well as through impairment of the cerebral vascular system. Therefore, given the VILI-related risk of further acute injury and chronic disability, it is imperative that a premature baby receive the safest respiratory support possible by initiating ventilation with an initial FiO2 of 21–30%, preferring NIV over endotracheal intubation, and if mechanical ventilation is required, avoid volutrauma by using a low VT.

## Data Availability

Not applicable.

## Abbreviations

GA: Gestational age
OS: Oxidative stress
VT: Tidal volume
VILI: Ventilation-induced lung injury
IL: Interleukin
CBF: Cerebral blood flow
NIV: Non-invasive mechanical ventilation
IPPV: Intermittent positive pressure ventilation
PEEP: Positive End-Expiratory Pressure
IVH: Intraventricular hemorrhage
PIP: Peak inspiratory pressure
NICU: Neonatal intensive care units
nCPAP: Nasal Continuous Positive Airway Pressure
HFNC: High-flow nasal cannula
NHFOV: High nasal frequency oscillatory ventilation
PLV: Limited pressure ventilation
VLV: Volume targeted ventilation
PVL: Periventricular leukomalacia
GV: Guaranteed volume
